# Prevalence of African animal trypanosomiasis among livestock and domestic animals in Uganda: a systematic review and meta-regression analysis from 1980 to 2022

**DOI:** 10.1038/s41598-023-47141-5

**Published:** 2023-11-20

**Authors:** Karla Rascón-García, Beatriz Martínez-López, Giuliano Cecchi, Caterina Scoglio, Enock Matovu, Dennis Muhanguzi

**Affiliations:** 1 Department of Medicine & Epidemiology, School of Veterinary Medicine, Center for Animal Disease Modeling and Surveillance (CADMS), University of California, Davis, USA; 2 Animal Production and Health Division, Food and Agriculture Organization of the United Nations, Rome, Italy; 3 Department of Electrical and Computer Engineering, Kansas State University, Manhattan, USA; 4 Department of Biotechnical & Diagnostic Sciences (BDS), College of Veterinary Medicine, Animal Resources and Biosecurity, Makerere University, Kampala, Uganda; 5 Department of Bio-Molecular Resources and Bio-Laboratory Sciences, College of Veterinary Medicine, Animal Resources and Biosecurity, Makerere University, Kampala, Uganda

**Keywords:** Diseases, Epidemiology

## Abstract

African animal trypanosomiasis (AAT) is one of the major constraints to animal health and production in sub-Saharan Africa. To inform AAT control in Uganda and help advance along the progressive control pathway (PCP), we characterized AAT prevalence among eight host species in Uganda and explored factors that influence the prevalence variation between studies. We retrieved AAT prevalence publications (n = 2232) for Uganda (1980–2022) from five life sciences databases, focusing on studies specifying AAT detection methods, sample size, and the number of trypanosome-positive animals. Following PRISMA guidelines, we included 56 publications, and evaluated publication bias by the Luis Furuya-Kanamori (LFK) index. National AAT prevalence under DNA diagnostic methods for cattle, sheep and goats was 22.15%, 8.51% and 13.88%, respectively. Under DNA diagnostic methods, *T. vivax was* the most common *Trypanosoma* sp. in cattle (6.15%, 95% CI: 2.91–10.45) while *T. brucei* was most common among small ruminants (goats: 8.78%, 95% CI: 1.90–19.88, and sheep: 8.23%, 95% CI: 4.74–12.50, respectively). Northern and Eastern regions accounted for the highest AAT prevalence. Despite the limitations of this study (i.e., quality of reviewed studies, underrepresentation of districts/regions), we provide insights that could be used for better control of AAT in Uganda and identify knowledge gaps that need to be addressed to support the progressive control of AAT at country level and other regional endemic countries with similar AAT eco-epidemiology.

## Introduction

African animal trypanosomiasis (AAT), a high-impact, wasting livestock disease caused by Trypanosoma species (*Trypanosoma* sp.) transmitted by tsetse flies and tabanids (*T. vivax*), is a major animal health constraint that impedes sustainable crop-livestock agriculture integration across 38 countries in sub-Saharan Africa (SSA). There have been several estimates of the economic impact of AAT in sub-Saharan Africa. As a baseline, without looking at price effects, Kristjansen et al.^1^ estimated that if an effective vaccine existed an extra USD 700 million of meat and milk could be produced. If producer and consumer surpluses were estimated, that is including gains to producers from cheaper production costs and to consumers from lower prices, Kristjansen et al., estimated that this would rise to USD 1300 million. The highest estimate to date (Budd^2^) was also based on estimating consumer and producer surplus, alongside the hypothesis that if tsetse were absent, cattle populations in the affected regions could double, generating an extra USD 4500 million from milk and meat production. More recently Abro et al.^3^ looked at the possible effects of the successful uptake of the waterbuck repellent blend technology and estimated that on a continental level, including consumer and producers’ surpluses, uptake of 50% could increase the value of meat and milk output by USD 900 million. Although it is an intermediate output, including the value of animal traction is likely to add 25–50% to that of meat and milk^2^.

Unlike progressive strides that have been made in human African trypanosomiasis (HAT) burden reduction over the past 20 years^4^, continent-wide efforts like the Pan-African Tsetse and Trypanosomiasis Eradication Campaigns (PATTEC) have not made comparable reduction in AAT burden. Reasons for this slow progress over the past two decades are many, but contributing factors include: the complexity of AAT epidemiology (i.e., the range of vectors, role of wild and domestic hosts, and the array of Trypanosoma species involved), dearth of financial and human resources, lack of robust control tools (i.e., no available vaccines, long outdated drugs, sub-optimal vector control tools, and no viable point-of-care diagnostics). Moreover, the absence of strong surveillance systems, poor strategic planning, and low awareness levels of decision-makers, donors and national veterinary authorities further exacerbate the challenge^5,6^.

The Progressive Control Pathways (PCP) are staged, strategic approaches for burden reduction or elimination, tailored to specific diseases of interest and adapted to the epidemiologic scenario unique to the targeted nation or region^7,8^. In 2017, the PCP was adopted for AAT, to improve control outcomes by optimizing the way programs are planned and implemented^5^. In particular, the PCP aims to help endemic countries set realistic, measurable targets for stepwise progression in AAT control. At the national level, Uganda just like most AAT-endemic countries, could be considered in Pre-Stage 1 or in Stage 1 (the first two of six stages) of the AAT PCP. Being at these early stages implies that, while there may be an expressed national-level commitment to burden reduction and an understanding of AAT risk and impact, limited progress has been made in sustainable burden reduction and that an integrated data management system is lacking^9^. For example, Uganda just like many other AAT endemic countries at this stage lacks a centralized and harmonized aggregate repository of AAT occurrence and burden data, which is an essential tool for the planning and implementation of targeted AAT control programs. To bridge this gap, the Food and Agriculture Organization of the United Nations (FAO) is promoting initiatives to centrally collate, store, harmonize and analyze AAT and vector abundance geospatial data, both at the national and the continental level. The main goal of these initiatives is to establish information systems of disease and vector occurrence (i.e., the ‘atlases’), useful for guiding AAT management and control efforts. However, while the continental atlas is presently being finalized^6,10–12^ and numerous national atlases have been produced^13,14^ this approach has not been taken up yet by the competent organizations in Uganda.

**Figure 1:**
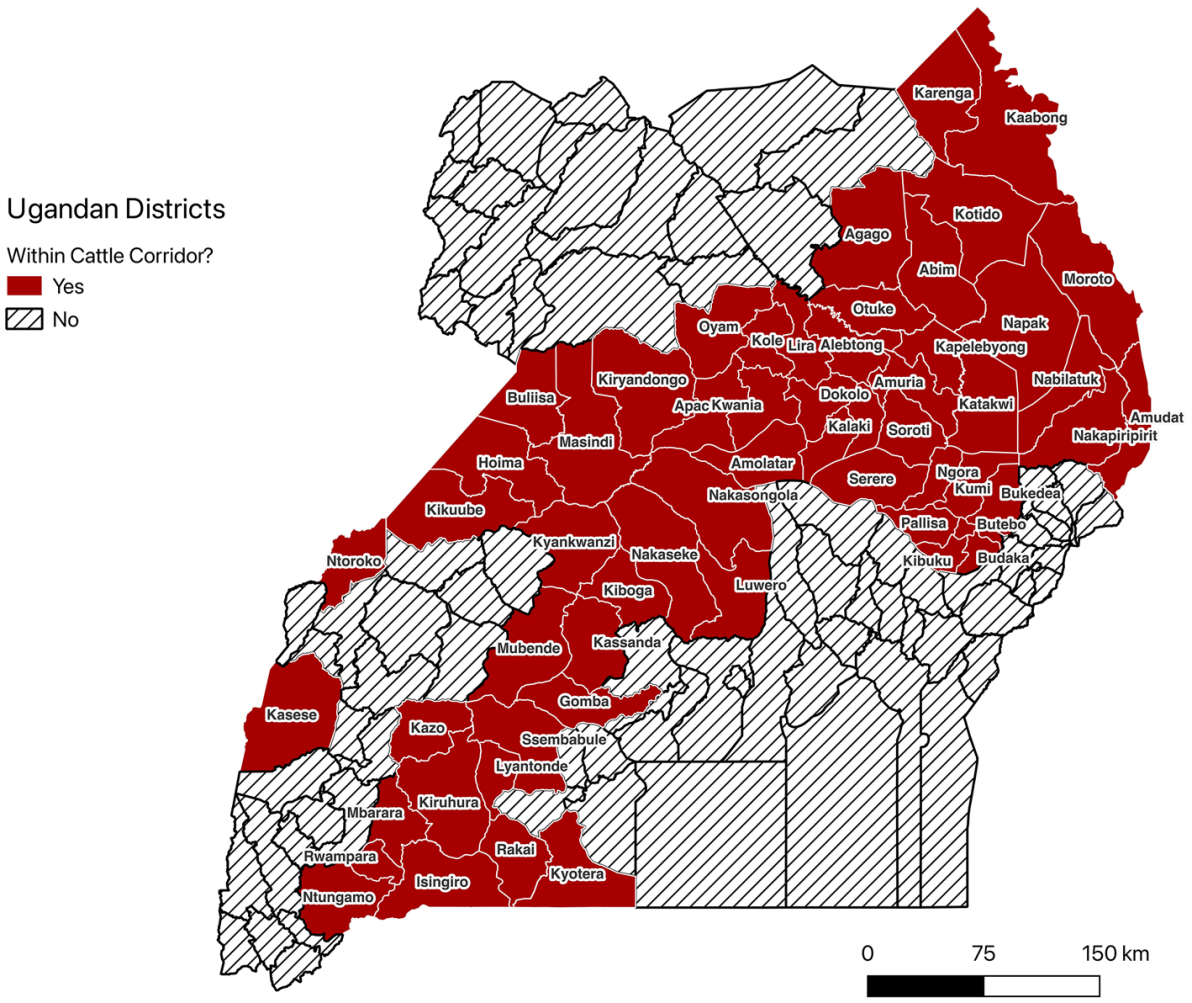
Map of Ugandan districts which constitute the Cattle Corridor (i.e., the subnational stretch where majority of livestock dwell) as described by the Uganda Bureau of Statistics^17,18^. The cattle corridor is constituted of districts with cattle density of > 50 head/square Km.

Prior to this study, previous meta-analyses investigated bovine, porcine, and small ruminant AAT^15,16^ at national level for several African countries. However, to the best of our knowledge granular AAT prevalence estimates (i.e., district-level information) for Uganda are unavailable. Consequently, national efforts to design and implement AAT surveillance and control programs have been developed in the absence of a clear description of AAT prevalence at high spatial resolution. National estimates cover a diverse topographic, ecologic, and agricultural landscapes, making the development of effective AAT intervention strategies at the local level challenging. Moreover, without accounting for the spatial distribution of livestock (i.e., cattle corridor) in the nation (see Fig. 1), efforts to control AAT burden will remain undermined.

In this study, we obtained published AAT prevalence estimates in multiple livestock and domestic animals over the past 42 years and conducted meta-analyses, generating more granular, as well as national-level, AAT prevalence estimates—estimates which cover diverse topographic and agricultural landscapes and now AAT estimates that will be useful for rationalizing AAT control through the PCP approach. We identified important gaps finding subnational variations in AAT prevalence in addition to finding that most districts within the cattle corridor lack published data. These results provide a foundation to better support subnational control programs as well as highlight the need for prevalence studies in districts for which no published AAT prevalence estimates are available.

## Methods

### Search strategy and application programming interfaces (APIs)

**Figure 2:**
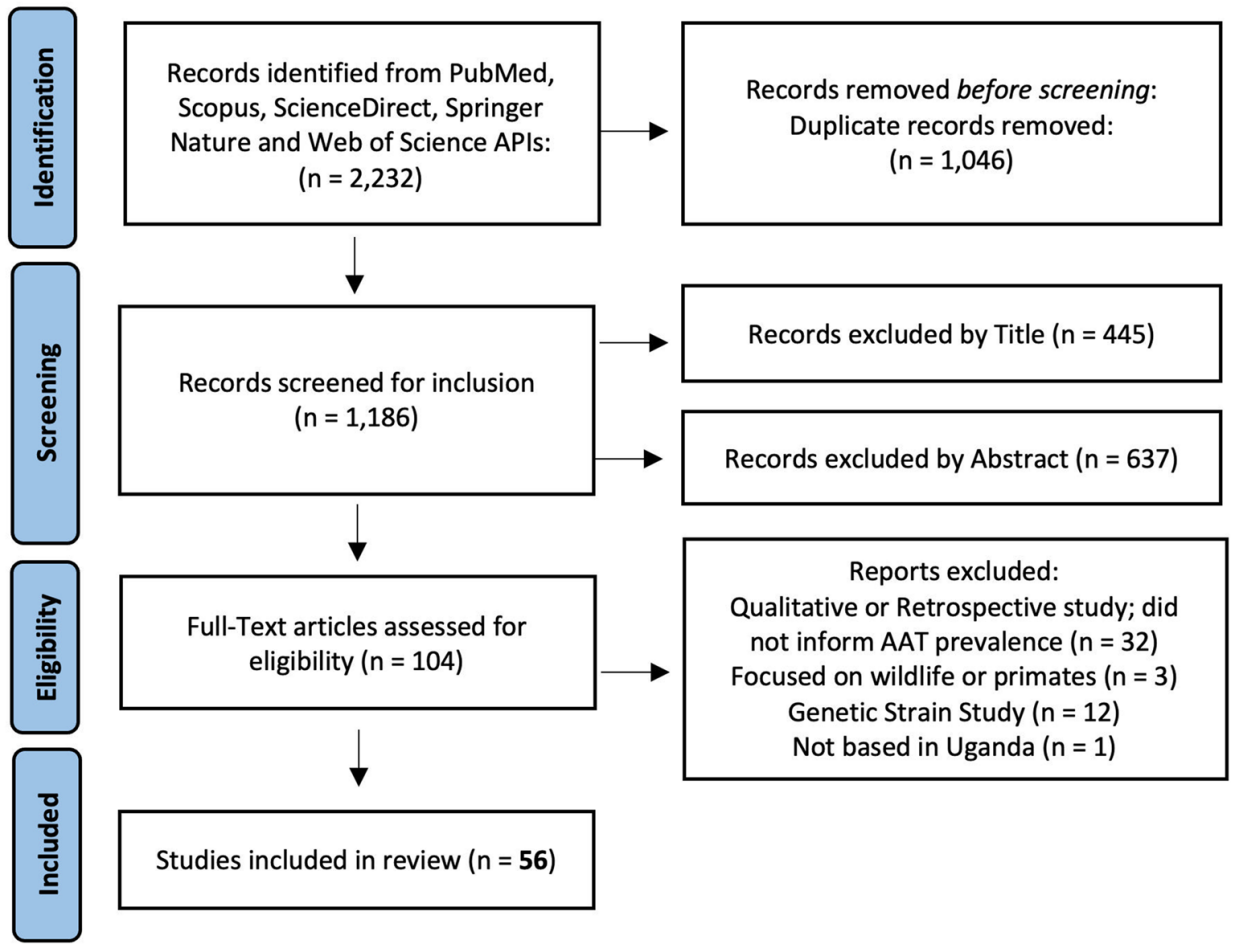
Flowchart depicting the record selection and article inclusion/exclusion process.

This study was conducted in accordance with the Preferred Reporting Items for Systematic Reviews and Meta-Analyses (PRISMA) guidelines^19^. A flow-chart depicting the selection process is presented in Fig. 2 and PRISMA checklist is provided in Supplementary Information. Articles published on PubMed, Scopus, ScienceDirect, Springer Nature and Web of Science databases were searched and retrieved through each database’s application programming interface (API). APIs allow for the two-way communication between systems and end-users for the retrieval of resources or objects^20^. In this study, the batch retrieval of peer-reviewed article information by use of standard query language was implemented. Following the automated retrieval of records, one team member (first author of this paper) worked independently screening article, conducting full-text evaluations, and extracting data from the included articles in accordance with the PRISMA flow described in the Fig. 2. Search terms across all databases included the following six queries: “Animal African trypanosomiasis (AAT) AND Uganda”, “trypanosomiasis AND Uganda”, “diagnostic test animal trypanosome AND Uganda”, “nagana AND Uganda”, “trypanosome risk factors AND Uganda” and “trypanosome prevalence AND Uganda.” Records were retrieved through Python modules PyMed^21^ and Elsapy^22^ for PubMed, Scopus, and Science Direct databases. Springer Nature and Web of Science APIs were accessed through direct URL requests^23,24^.

### Inclusion/exclusion criteria

The articles included in this study had to be from Uganda, written in English, and published between 1980 and 2022. Moreover, full-text documents needed to be available in addition to their reporting of each study’s sample size, number of trypanosome-positive animals (specifically, African trypanosomes), and diagnostic tool used for testing. Articles were excluded if they: focused on *Trypanosoma cruzi* infections, involved experimental infections, were lab-based, or involved animals with a non-agricultural importance (e.g., primates, wild birds, etc.). Articles reporting prevalence of multiple diseases were considered contingent on the requirement that Trypanosome-specific natural infections were reported for all host species involved.

### Publication quality assessment

Munn et al.^25^ developed a specific critical appraisal checklist for evaluating studies reporting prevalence data. Using this checklist, we applied the same tool as implemented in a previous bovine trypanosomiasis meta-analysis focused on selected African countries^26^. The adapted tool included all original questions except the question, “Was the response rate adequate, and if not, was the low response rate managed appropriately?” (see Supplementary Information). Possible responses during this appraisal were “yes,” “no,” or “unclear,” where responses were coded “2,” “0,” and “1,” respectively.

### Publication bias

Articles are more likely to be published if significant results are observed or otherwise deemed relevant. Moreover, studies with small sample sizes or issues with precision may lead to their exclusion from publication even if they present relevant information. Generalization and validity of results can pose serious problems if this publication bias isn’t explicitly evaluated^27^. Common approaches for testing publication bias include Egger’s regression tests^28^, visual examinations of funnel-plots, the Luis Furuya-Kanamori (LFK) index^29^, and Vevea and Hedges weight-function model^30^, among others. Visual assessment of funnel plot asymmetry can be very subjective. Consequently, we used the LFK index to evaluate publication bias by species-specific analyses. The LFK values more extreme than $\pm$ 2 indicate the presence of publication bias as indicated by “major asymmetry” in the observed funnel plot. Together, funnel plot visualizations and LFK index values were both used to assess publication bias.

### Data extraction

For each article included in analyses, the host species, number of animals sampled, number that tested positive, diagnostic test used and *Trypanosoma* sp. detected were all extracted from articles. The village, subcounty, parish, and/or district where the animals were sampled from was additionally recorded as well as the year the study was conducted. Diagnostic methods used in each study were classified into one of three “*target*” categories according to diagnostic techniques as summarized by Desquesnes et al.^31^. Diagnostic techniques were classified into three groups depending on their target: antibodies, DNA, or visualization of the entire *Trypanosoma sp*. in blood smears (thin and thick) or the buffy coat.

### Data analysis

Heterogeneity (between-study variation) was estimated by a random-effects model using a Freeman-Tukey Double arcsine transformation for estimating overall proportions^32^. Heterogeneity was analyzed by host species except in cases where only one article was published for host species (i.e., camels, donkeys, and chickens). In all other analyses, between-study variance was quantified using the inverse variance index statistic (I^2^ statistic), as previously described by Higgins et al.^33^ and significance assessed using the Cochran Q test^34^. Heterogeneity values range from 0 to 100%, where values of 0% suggest no between-study variations—all variation would otherwise be explained by sampling error, or within-study variations.

Sub-group analyses were conducted for each species, stratifying by district and diagnostic target to capture district level AAT prevalence estimates by host category. Prevalence was further analyzed by region (Central, Eastern, Northern, and Western), study period (1980–1999, 2000–2009, 2010–2022), and study sample sizes. The number of animals sampled varied widely by species. Consequently, different categorization systems were used across species. For cattle and pigs, sample sizes were grouped into three classes: $\le$ 500, 501–1500, and > 1501. Similarly, dogs [$\le$ 50, 51–149, and > 150] and goats [$\le$ 300, 301–999, and > 1000] were grouped into three classes. With minimal variation, sheep were the only host species with study sample size grouped into two levels: $\le$ 100 and > 101. Group analyses were not possible for dogs, donkeys, and camels for which only one article was available. Finally, the prevalence of trypanosome species detected was stratified by diagnostic target for each host species.

Forest plots were generated for each host species, visually displaying prevalence estimates by diagnostic technique. For spatial mapping of AAT prevalence by district, Jenks natural breaks were used to create maps with categories of either 3 or 5 classes depending on the observed range in prevalence estimates host category.

### Meta-regression

To identify sources of heterogeneity, univariate and multivariate meta-regressions were conducted with district, study period, sample size, and diagnostic target fitted as independent variables. For articles where the study year was not reported, data were assumed to have been collected one year prior to the publication date. Explanatory variables with a p-value < 0.25 in univariate analyses were subsequently included in multivariate meta-regressions. Multivariate meta-regressions were conducted to ascertain the amount of heterogeneity explained by multiple variables. All analyses were conducted using R version 4.2.2, with the primary packages used including *meta*^35^ and *metasen*s^36^.

## Results

### Literature search

A total of 2234 records were retrieved using their APIs across all five databases under the six search queries listed. Duplicate records (either because of duplicity across databases and/or duplicate returns under different search queries) were excluded (*n* = 1046) resulting in 1186 unique records which were available for screening. After title and abstract inspection, 104 articles were selected for full-text evaluation, of which, 56 unique articles satisfied inclusion criteria and were therefore incorporated in analyses (see Supplementary Table S1).

### Study quality assessment

Using Munn et al.^25^ appraisal tool, article mean scores ranged from 1.50 to 2.00 (see Supplementary Information). Most common low-scoring criteria pertained to (1) whether animals were recruited in an appropriate fashion or (2) whether the study’s sample size was justified or sufficed. Of the 56 articles included, 37.5% (*n* = 20) failed to clarify a sampling method, animal recruitment criteria or were otherwise unclear. Similarly, 71.4% (*n* = 40) of the 56 articles didn’t explicitly conduct a sample size calculation or otherwise failed to justify the sample size used. By our assessment, all articles (n = 56) were of acceptable quality.

### Meta-analysis of AAT prevalence

The 56 articles that were included in the analyses drew their animal samples from 48 of the 146 districts of Uganda^34^. Eight host species were represented, with the most published species being cattle (n = 48), followed by pigs and goats (10 and 8 articles, respectively) (see Table 1). Only one record reporting AAT prevalence in camels, chickens, and donkeys was retrieved. Four were identified related to both canine and ovine trypanosomiasis. Some articles included multiple species in their studies, hence article counts do not add up to 56 unique records.

**Table 1:** National AAT prevalence by host species by diagnostic method used.

Host	Diagnostic target	No. studies	Positive	Total samples	% Prevalence (95% CI)	I^2^	*p*-value
Camels	Parasite	1	73	112	65.18 (56.08–73.76)	–	1.00
Cattle	Antibodies	4	1008	2830	27.27 (8.58, 51.58)	99.3	< 0.0001
	DNA	23	5254	28,922	22.15 (15.87, 29.14)	99.4	< 0.0001
	Parasite	32	4300	32,171	13.36 (10.33, 16.7)	98.6	< 0.0001
Chickens	DNA	1	6	77	7.79 (2.68–15.01)	–	1.00
Dogs	DNA	1	14	113	12.39 (6.88, 19.17)	–	1.00
	Parasite	4	9	417	1.71 (0.01, 5.22)	68.7	0.0225
Donkeys	DNA	1	26	71	36.62(25.74–48.21)	–	1.00
Goats	DNA	3	197	1485	13.88 (1.83, 34.31)	98.9	< 0.0001
	Parasite	7	33	2516	1.07 (0.11, 2.75)	88.3	< 0.0001
Pigs	DNA	4	183	1287	13.54 (2.46, 31.32)	98.4	< 0.0001
	Parasite	9	389	4911	10.50 (4.62, 18.26)	98.1	< 0.0001
Sheep	DNA	2	30	306	8.51 (3.81, 14.70)	42.7	0.19
	Parasite	4	35	471	6.84 (0.96, 16.70)	89.1	< 0.0001

In addition to sampling multiple hosts, some studies collected samples from more than one district. Nine studies reported prevalence as an aggregate across all sampled districts and failed to either specify the denominator (i.e., the sample size associated with each district) or stratify the numerator (i.e., number of trypanosome positive animals) for each district. Corresponding authors to these nine articles were contacted directly, of which responses were able to reconcile two articles. Attributes of articles included in analyses are detailed in Supplementary Table S1.

### National AAT prevalence by animal species

Across all 56 articles included in analyses, 11,557 of 75,689 animals sampled were positive for at least one *Trypanosoma* species. National AAT prevalence estimates for each host species by diagnostic target are presented in Table 1.

To account for diagnostic performance characteristics, AAT prevalence across districts was sub-analyzed by diagnostic technique and provided in Supplementary Table S2. Forest plots estimates for ruminants can be found in Fig. 4 and forest plots for all remaining host species are provided in Supplementary Fig. S3.

### AAT prevalence by district—cattle and small ruminants

**Figure 3:**
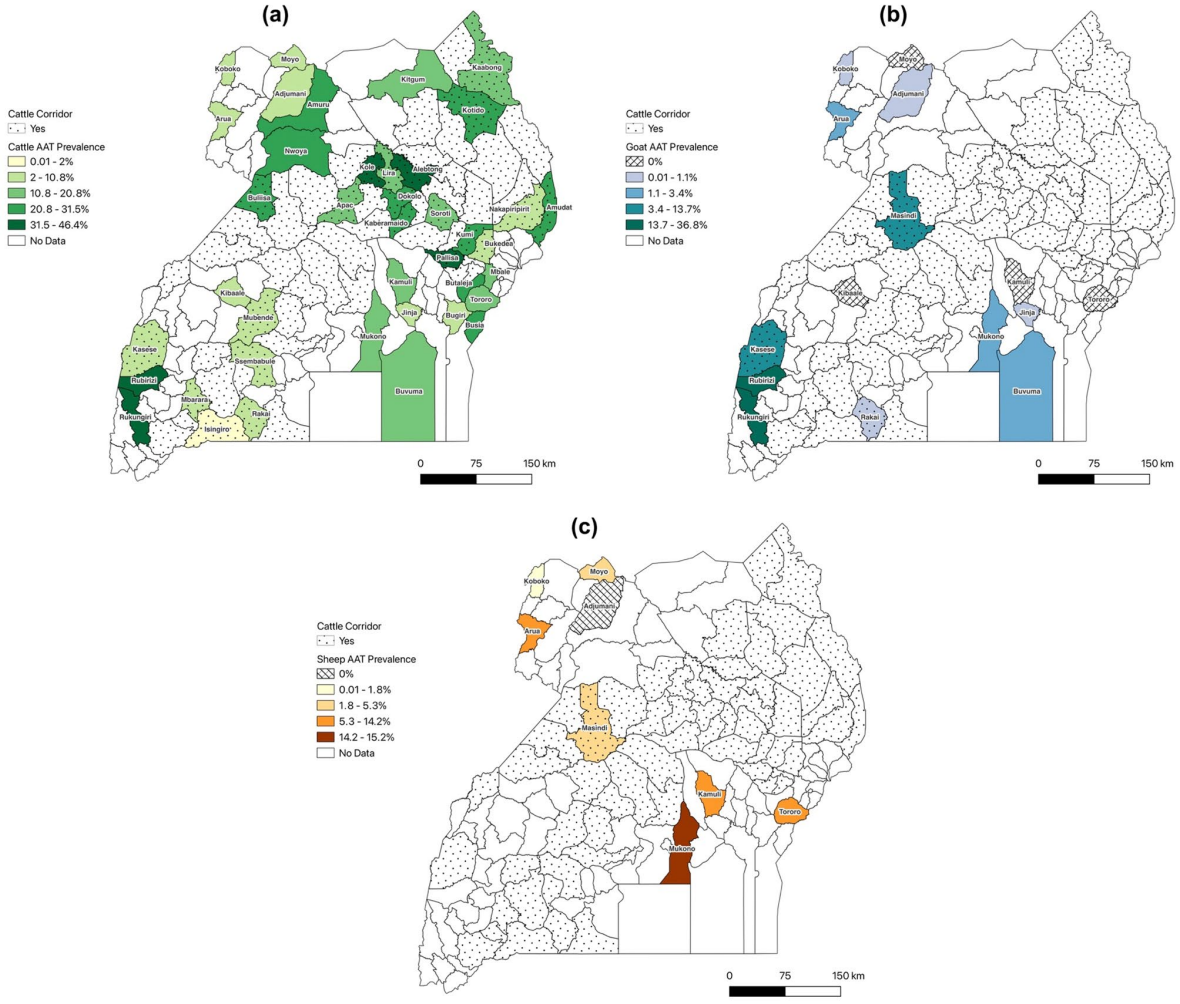
AAT prevalence by district, as estimated by all diagnostic methods for (**a**) cattle and small ruminant (**b**) goats and (**c**) sheep.

**Figure 4:**
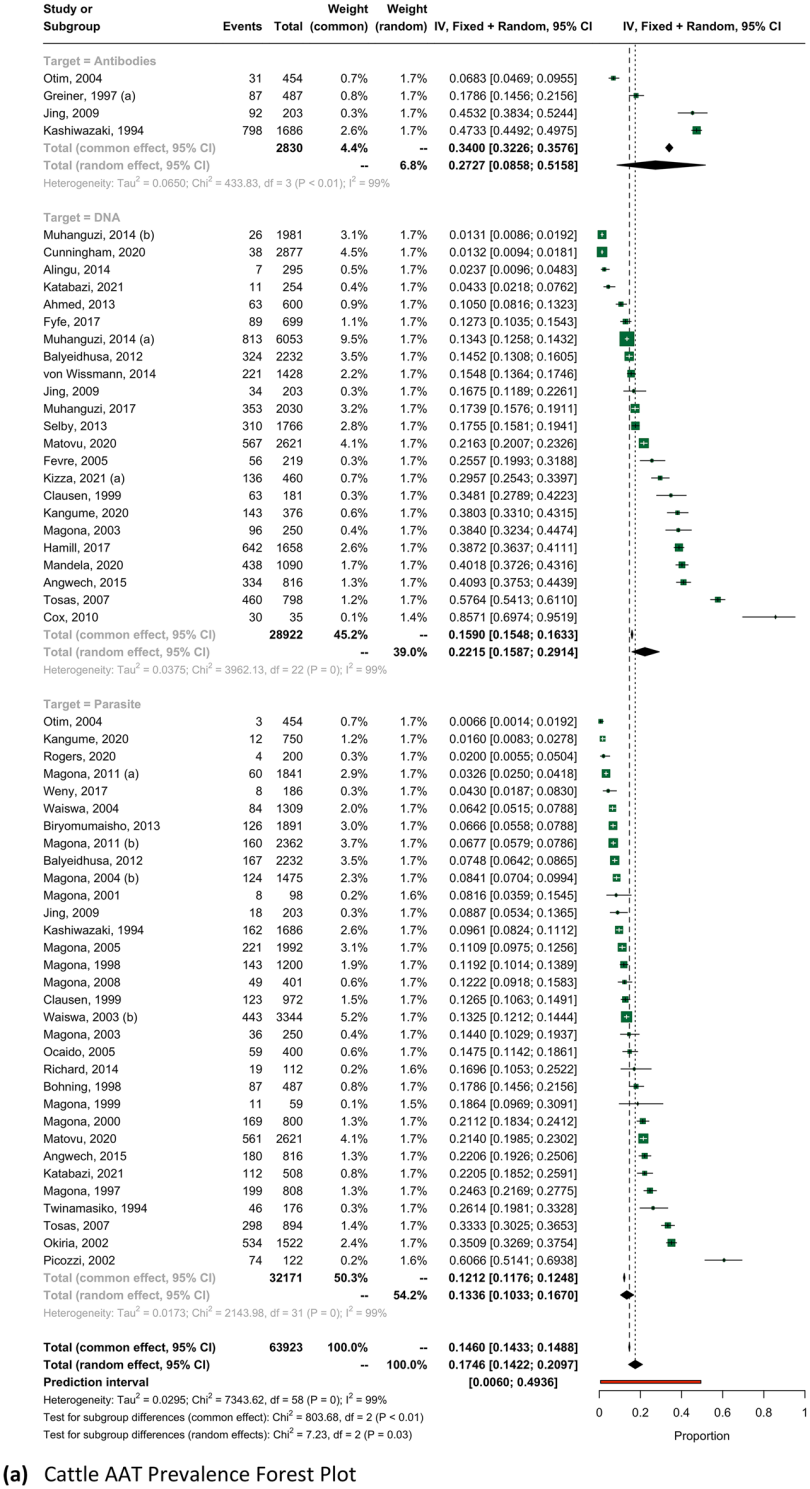

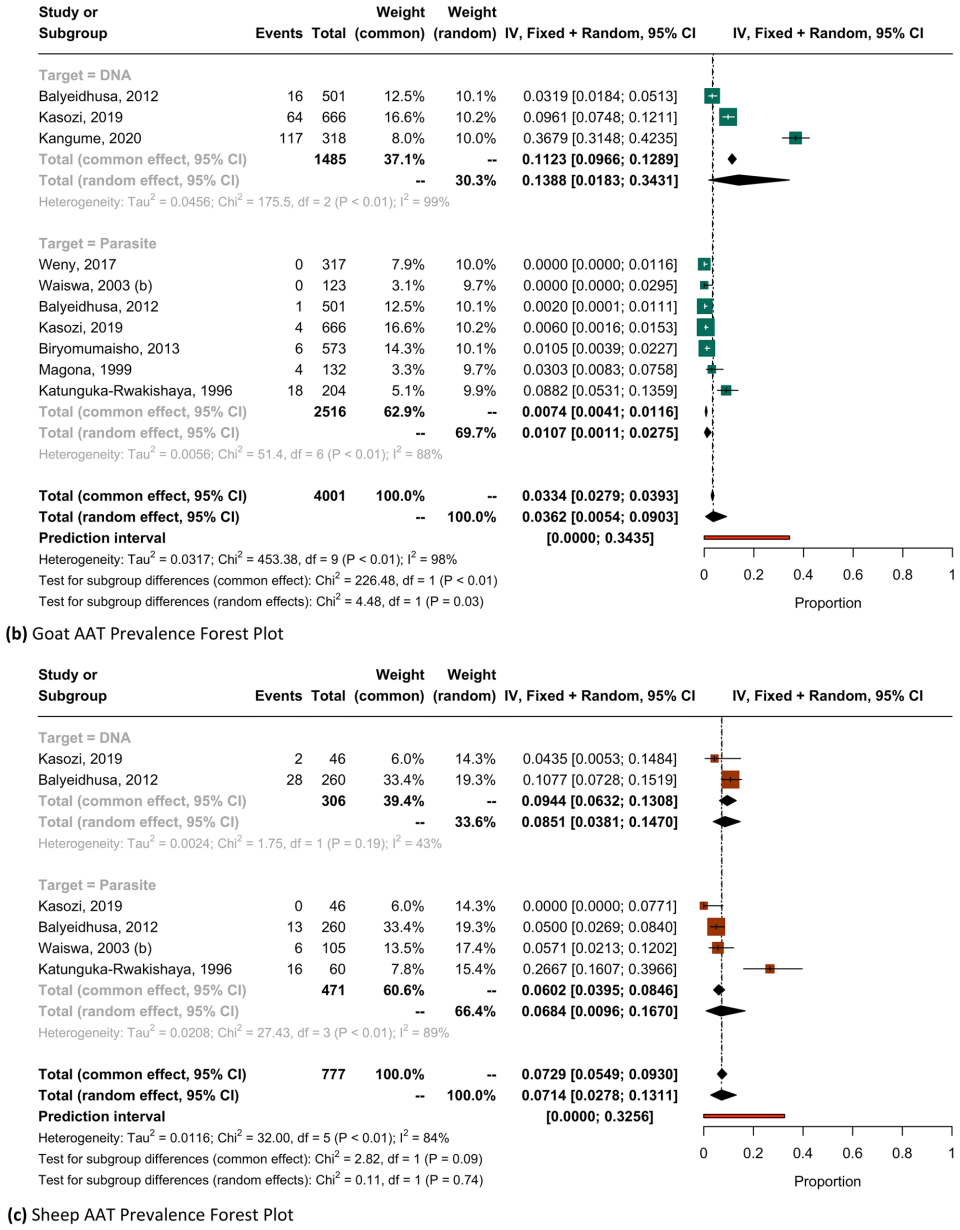
AAT Prevalence Forest Plots for (**a**) cattle, (**b**) goats, and (**c**) sheep. NOTE: Forest plots in these figures report prevalence as a value between 0.0 and 1.0.

Jenks natural breaks with five classes were used to produce maps provided in Fig. 3. Forty districts were sampled for bovine trypanosomiasis, all of which detected at least one positively infected animal. Eight and fifteen districts were sampled for ovine and caprine trypanosomiasis, respectively. AAT prevalence by host-species was sub-analyzed by diagnostic technique with ruminant estimate forest plots presented in Fig. 4.

### AAT prevalence by district—camels, chickens, and donkeys

Only one article was eligible for characterizing each camel, chicken, and donkey AAT prevalence. Chicken and donkey AAT articles included samples from more than one district. Jenks natural breaks with three classes were used for these articles except for camel AAT, which involved a single study from Moroto district (Supplementary Fig. S1). Though meta-analyses could not be formally conducted for these species, their estimates were worthy of mentioning in this study.

### AAT prevalence by district—dogs and domestic pigs

Eleven and twelve districts were sampled for canine and porcine trypanosomiasis, respectively. Only diagnostic tests that targeted direct parasite detection or DNA detection were used. Canine (Supplementary Fig. S2a) and porcine (Supplementary Fig. S2b) prevalence studies retrieved involved detection of *T. brucei*, *T. vivax*, and *T. congolense* in at least one dog and pig across the study districts (Also see Supplementary Table S3).

### AAT prevalence by sub-groups

Results for subgroup analyses are presented in Supplementary Table S3. In some instances, studies reported results from samples taken during different years and/or using different diagnostic techniques. Consequently, the number of studies recorded in Supplementary Table S3 don’t always add up to the total number of studies listed in Table 1. DNA target tools estimated the highest prevalence of AAT across all host species. Cattle represent the highest pooled AAT prevalence [22.15%] under DNA detection tools followed by goats, pigs, and dogs with pooled national estimates of 13.88%, 13.54%, and 12.39% respectively.

### AAT prevalence by geographical regions of Uganda

Bovine AAT prevalence, under all diagnostic methods, is estimated to be highest in the Northern and Eastern regions (19.26% and 17.84%, respectively) compared to the Central and Western regions (12.69% and 7.08%, respectively). A table of results is available in Supplementary Table S5. Small ruminant AAT prevalence estimates similarly suggest subnational variations with caprine AAT highest in the Western region (6.27%) and ovine AAT highest in the Central region (15.23%, see Supplementary Table S6). Though few studies investigated AAT among dogs, just as with bovine AAT, the Northern region estimated the highest canine AAT prevalence (7.96%) while the Central region estimated a considerably higher porcine AAT prevalence (30.62%, see Supplementary Table S7).

### Publication bias

Publication bias could not be evaluated for donkeys, camels, and chickens as only one article was found for each host species. Significant publication bias with a 3.05 LFK index value was estimated for porcine trypanosomiasis. Minor bias was estimated for dog and cattle prevalence data (1.12 and − 0.9 LFK, respectively) with goat and sheep publications suggesting minimal bias (0.14 and 0.26 LFK, respectively). Funnel plots of bias are provided for all host species in Supplementary Fig. S4.

### Meta-regressions

Meta-regressions were only conducted for host species that had more than one article, which excluded camels, chickens, and donkeys from analyses. District, sample size, and diagnostic target were significant predictors of bovine trypanosomiasis in univariate meta-regression models (p < 0.25) with district influencing heterogeneity the most (14.93% of variation). For all species except goats and domestic pigs, the national district explained the most heterogeneity with 39.05% of canine trypanosomiasis explained by district and 55.78% of variation of ovine trypanosomiasis (Table 2).

**Table 2:** Univariate meta-regression analysis results.

Host	Variable	*p*-value	R^2^ (%)
Camels	–	–	–
Cattle	District	**0.043**	14.93
	Study period	0.40	0.00
	Sample size	0.074	0.00
	Diagnostic target	**0.0034**	0.03
Chickens	–	–	–
Dogs	District	0.099	39.05
	Study period	0.97	0.00
	Sample size	**0.029**	26.82
	Diagnostic target	**0.045**	23.42
Donkeys	–	–	–
Goats	District	0.157	0.00
	Study period	**0.046**	0.00
	Sample size	0.88	0.00
	Diagnostic target	** < 0.0001**	43.05
Pigs	District	0.84	0
	Study period	0.064	22.57
	Sample size	0.27	0
	Diagnostic target	0.067	22.19
Sheep	District	**0.049**	55.78
	Study period	0.058	26.21
	Sample size	0.21	10.55
	Diagnostic target	0.66	0.00

p-values in bold indicate models with a p-value < 0.05.

Different combinations of significant univariate predictors were assessed to evaluate their combined effect on AAT prevalence. Interactions could not be modeled in two instances for dogs and in one instance for goats due to low observations. Where interactions could not be modeled, the largest additive model was still analyzed and presented (Table 3).

**Table 3:** Multivariate meta-regression analysis results.

Host	Model	*p*-value	R^2^ (%)
Camels	–	–	–
Cattle	District * Sample Size	0.059	0.94
	District * Target	0.056	7.81
	Sample Size * Target	**0.013**	0.00
	District * Target * Sample Size	0.43	0.00
Chickens	–	–	–
Dogs	District * Sample Size	0.099	39.05
	District + Target	**0.0079**^†^	71.69
	Sample Size * Target	0.056	33.63
	District + Sample Size + Target	**0.0079**^†^	71.59
Donkeys	–	–	–
Goats	District * Study Period	0.18	0.00
	District * Target	** < 0.0001**	59.52
	Study Period * Target	** < 0.0001**	66.77
	District + Study Period + Target	** < 0.0001**^†^	54.75
Pigs	Target*Study Period	< 0.0001	73.34
Sheep	District * Study Period	**0.0029**	73.50
	District * Sample Size	**0.0029**	73.50
	Study Period * Sample Size	**0.0229**	47.99
	District * Study Period * Sample Size	**0.0029**	73.50

Note: an asterisk (*) denotes an interaction between the two neighboring variables, while a plus symbol (**+**) denotes an additive effect model where interactions could not be executed.

p-values in bold indicate models with a p-value < 0.05.

^†^Could not run interactions—the number of parameters estimated exceeded the number of observations.

Study period, district, and diagnostic target explained most of AAT prevalence variations in different combinations. Cattle AAT prevalence was best explained (7.8%) by district and diagnostic target, jointly. This low *R*^*2*^ indicates that factors outside of those analyzed here contribute to cattle AAT. Diagnostic target and study period jointly explained a considerable proportion of porcine prevalence (73.3%). District and study period explained a similar proportion of ovine trypanosomiasis (73.5%) while study period and diagnostic target best explained 66.8% of caprine trypanosomiasis.

## Discussion

Efforts to progressively reduce and consequently eliminate AAT as an animal health problem warrant a subnational detailed description of its prevalence and distribution. Our study provides the most comprehensive information about AAT prevalence in camels, cattle, chickens, dogs, donkeys, goats, pigs, and sheep at fine spatio-temporal scales in Uganda. Although AAT is not considered to be a major threat to the global north, nor does it affect international livestock trade^9^ it has a heavy impact on smallholder livestock production systems in rural Sub-Saharan Africa. For efforts using frameworks like the PCP to be successful, intervention planning and surveillance decisions must be based on detailed and reliable data. Previous meta-analyses have made strides to fill information gaps by investigating bovine, porcine and small ruminant AAT at national level for select African countries^15,16^. With a focus on agricultural livestock health, our work goes one step further by providing a comprehensive, more granular AAT picture specific for Uganda. We obtained AAT prevalence estimates in domestic animals of economic and agricultural importance to the nation and subnational described AAT over diverse topographic and agricultural landscapes.

Diagnostic techniques used across studies were categorized into three general diagnostic target categories (i.e., DNA, Antibodies, or Parasite) according to Desquesnes et al.^31^. Accounting for diagnostic performance characteristics was imperative for reliable results as sensitivity and specificity differ by the diagnostic tool used. Moreover, the reliability of diagnostic tools can be seen to vary depending on the time of diagnosis as it relates to the course of an infection. Direct parasite detection is limited in sensitivity (14–24%) and specificity, underestimating the true prevalence^31^. On the other hand, DNA and antibody detection methods are more sensitive with ITS1-PCR sensitivities ranging between 54 and 75% and ELISA (against whole antigens) sensitivities estimated at 90.5+ %^31^, yielding more reliable estimates. Only studies focused on bovine trypanosomiasis reported the use of antibody detection methods while all others reported prevalence by both parasite and DNA detection methods. All diagnostic techniques indicated high AAT prevalence among cattle (antibody detection: 27.27%; DNA detection: 22.15%; parasite detection: 13.36%), domestic pigs (DNA detection: 13.54%; parasite detection: 10.50%), sheep (DNA detection: 8.51%; parasite detection: 6.84%), and goats (DNA detection: 13.88%; parasite detection: 1.07%) in descending order. As expected, targeting parasite genetic material and antibodies were associated with the highest sensitivity^31^. Seven *Trypanosoma* sp. were detected among eight animal hosts evaluated. As such, *T. vivax* was the most prevalent trypanosome species detected among cattle (12.31%) and pigs (12.38%) using molecular methods. Additionally, *T. brucei* the was most detected *Trypanosoma* sp. among goats (8.78%), sheep (8.23%) and dogs (8.85%) using DNA-target methods. These results affirm that AAT is still a major constraint to animal health and production in Uganda with *T. vivax* that is both cyclically and mechanically transmitted^37^ being the commonest *Trypanosoma* sp. for cattle and pigs, while the more chronic *T. brucei* is common in small ruminants and dogs.

Across DNA-target methods, cattle accounted for the highest ruminant national prevalence with a high average of 22.15% under DNA target methods, followed by goats and sheep with moderate prevalence estimates of 13.88% and 8.51%, respectively. The higher cattle AAT prevalence could potentially be explained the tsetse fly preference to feed on cattle and humans^38^ rather than on other ruminants. As has previously been advised^39,40^, spraying cattle with tsetse effective insecticides (live baits) after they have been treated with trypanocidal drugs should be advocated for to significantly reduce ruminant AAT prevalence in Uganda. However, small ruminants should also be included in AAT control programs in situations where financial and human resources can be extended beyond cattle populations in endemic settings.

Trypanosomiasis among non-ruminants depict a high AAT prevalence in domestic pigs with a national average of 13.54% under DNA-target methods. Though AAT among camels, chickens, and donkeys were not formally analysed, we considered it important to mention these host species and describe their apparent prevalence because, though they may not play a large economic role in Uganda’s livestock sector, their presence in the country may still poses them as potential reservoirs for both human and animal trypanosomiasis. Donkeys and camels are important ways of transportation in rural North-eastern Uganda. They are therefore important in the eco-epidemiology of AAT and should therefore be considered in AAT surveillance and control programs^41^.

At subnational level, bovine AAT prevalence by molecular methods was highest in the northern (19.26%) and eastern (17.84%) parts of the country. The northern region was affected by the Lord’s Resistance Army (LRA) war from 1989 to mid 2000; during which time there were no major AAT control activities implemented. When the LRA war ceased during the year 2006, the northern region districts were then restocked by livestock from AAT endemic eastern region, further aggravating the AAT prevalence^42,43^. The lack of sustainable AAT control programs in the eastern region^44^, disruption of economic activities and AAT control programs, widespread turmoil caused by the LRA war, as well as livestock restocking from AAT endemic regions after the war created just the right milieu for insidious AAT transmission to thrive in the northern and eastern regions. Conversely, the shift from pastoral to more intensive livestock production systems in southwestern Uganda during the mid 1980’s (driven by the need to increase milk production), came with intensive insecticide applications. The intensive application of deltamethrin insecticide for tick control in dairy production systems in southwestern Uganda has resulted in reduced tsetse and biting fly density as well as a reduced AAT prevalence, explaining the moderate AAT prevalence by molecular techniques (7.08%) in this region compared to the rest of the country. Additionally, the AAT prevalence for southwestern Uganda is likely to be an underestimate of the true prevalence given that considerably fewer studies have been conducted across districts in this region compared to those in northern and eastern Uganda.

Of the 40 districts where cattle were sampled, less than half (*n* = 13) represented districts with the highest livestock density (i.e., the cattle corridor), highlighting an urgent need to screen livestock in the rest of the cattle corridor districts for which there are no published AAT prevalence data. Cattle in Kole and Alebtong districts (situated well within the corridor) estimate the highest AAT prevalence in the Northern Region (> 30% prevalence). Subnational characterizations of goat and sheep AAT prevalence estimates were, once again, limited due to fewer studies that focus on small ruminant trypanosomiasis. When looking at estimates by district, however, goat AAT was highest in western districts Rubirizi and Rukungiri (36.79% prevalence across both districts). Ovine trypanosomiasis, on the other hand, was highest in Mukono district (15.23%) of the Central region bordering the Kampala district. These data reflect the districts where goats were sampled and not where the prevalence of small ruminant AAT might be highest.

There are several limitations in this study. Most of the publications included (71.4%) either did not conduct sample size calculations or otherwise failed to explicitly justify their number of recruited animals. Additionally, we aimed to describe prevalence at the district level, but our results found that not all districts in Uganda have been sampled. Moreover, most studies focused on bovine AAT, limiting our ability to characterize the full extent of AAT across the larger livestock landscape. Nevertheless, these findings corroborate current literature which highlight *T. vivax, T. brucei* and *T. congolense* as the three *Trypanosoma* sp. of greatest importance in the region^31,45–47^. By leveraging available APIs, we were not only able to collect large batches of data rapidly and systematically, but we were able to search widely and consider articles that might have otherwise been missed through manual searches. Our AAT prevalence estimates build on past AAT meta-analyses^15,16^ which kicked off the building of an AAT burden database for Uganda. The development of such a database can support the PCP approach to accelerate AAT burden reduction. The PCP ‘Below Stage 1’ and ‘Stage 1’ endeavors focus on creating the national-level institutional and technical environments for a stepwise progression; these early-stage activities encourage the generation of a database which should be collaboratively managed by governmental bodies with the possible support of academic institutions. Results from this work could contribute to the development of a national AAT Atlas in partnership and with the lead of Uganda’s national mandated authorities.

In any event, AAT prevalence data are missing from districts that are known to have high livestock densities. Cattle and small ruminants are known to comingle at grazing and watering points, yet small ruminant AAT is worryingly understudied. These results are helpful as they have determined districts with no published AAT data for the past 40+ years (the time window scope of this review). The identification of such districts can serve as the starting point for discourses focused on the design of risk-based, cost-effective AAT control and surveillance programs. For example, we could recommend interventions start with districts and regions currently characterized with high AAT prevalence. We hope these results can support AAT control by ideally leveraging the PCP framework, ultimately helping to move the country from early stages of the PCP (Pre Stage 1 and Stage 1) towards a more sustainable burden reduction state.

### Supplementary Information

Supplementary Information.

## Data Availability

The dataset analyzed in this study is available upon reasonable request from the corresponding author.
